# Conflicting genomic signals affect phylogenetic inference in four species of North American pines

**DOI:** 10.1093/aobpla/plw019

**Published:** 2016-04-08

**Authors:** Tomasz E. Koralewski, Mariana Mateos, Konstantin V. Krutovsky

**Affiliations:** 1 Department of Ecosystem Science and Management, Texas A&M University, 2138 TAMU, College Station, TX 77843-2138, USA; 2 Department of Wildlife and Fisheries Sciences, Texas A&M University, 2258 TAMU, College Station, TX 77843-2258, USA; 3 Department of Forest Genetics and Forest Tree Breeding, Büsgen-Institute, Georg-August University of Göttingen, Büsgenweg 2, D-37077 Göttingen, Germany; 4 N.I. Vavilov Institute of General Genetics, Russian Academy of Sciences, Moscow 119333, Russia; 5 Genome Research and Education Center, Siberian Federal University, 50a/2 Akademgorodok, Krasnoyarsk 660036, Russia

**Keywords:** *Australes*, drought tolerance, parallel evolution, phylogeny, plant defence, southern pines, wood formation

## Abstract

Adaptive evolutionary processes in plants may be accompanied by episodes of introgression, parallel evolution and incomplete lineage sorting that pose challenges in untangling species evolutionary history. Genus *Pinus* (pines) is one of the most abundant and most studied groups among gymnosperms, and a good example of a lineage where these phenomena have been observed. Pines are among the most ecologically and economically important plant species. Some, such as the pines of the southeastern USA (southern pines in subsection *Australes*), are subjects of intensive breeding programmes. Despite numerous published studies, the evolutionary history of *Australes* remains ambiguous and often controversial. We studied the phylogeny of four major southern pine species: shortleaf (*Pinus echinata*), slash (*P. elliottii*), longleaf (*P. palustris*) and loblolly (*P. taeda*), using sequences from 11 nuclear loci and maximum likelihood and Bayesian methods. Our analysis encountered resolution difficulties similar to earlier published studies. Although incomplete lineage sorting and introgression are two phenomena presumptively underlying our results, the phylogenetic inferences seem to be also influenced by the genes examined, with certain topologies supported by sets of genes sharing common putative functionalities. For example, genes involved in wood formation supported the clade *echinata*–*taeda*, genes linked to plant defence supported the clade *echinata*–*elliottii* and genes linked to water management properties supported the clade *echinata*–*palustris*. The support for these clades was very high and consistent across methods. We discuss the potential factors that could underlie these observations, including incomplete lineage sorting, hybridization and parallel or adaptive evolution. Our results likely reflect the relatively short evolutionary history of the subsection that is thought to have begun during the middle Miocene and has been influenced by climate fluctuations.

## Abstract (precis)

Eleven nuclear genes and multiple phylogenetic techniques were used to study relationships among four of North American pines from subsection *Australes* (genus *Pinus*). Support for phylogenies reconstructed based on all data was generally weak and inconsistent among methods. Subsequent analysis revealed that certain topologies were supported by genes with common putative functionalities. Therefore, each set of genes was analysed independently. Recovered alternative topologies were highly supported and the results were consistent among methods. Multiple evolutionary hypotheses could potentially explain the observed patterns, although incomplete lineage sorting seems to be the simplest one.

## Introduction

Plant genome structure and evolution are subjects of intensive investigations with recent milestone advances spanning whole-genome sequencing of some of the largest plant genomes, such as those of pines and spruces (Birol *et al.* 2013; Nystedt *et al.* 2013; Krutovsky *et al.* 2014; Neale *et al.* 2014; Zimin *et al.* 2014). These advancements bring opportunities to ask more focussed questions about the dynamic processes that have contributed to plant genome evolution and influenced phylogenetic relationships among plant species. Duplications, from single gene to whole genome, are an intrinsic process that appears to be the major force driving genome evolution in plants (Freeling 2009; Li *et al.* 2015). Other processes, such as introgression, incomplete lineage sorting and parallel evolution, primarily influenced by extrinsic factors, additionally contribute to plant genome complexity and evolution (Martinsen *et al.* 2001; Wood *et al.* 2005; Willyard *et al.* 2009). The latter processes may also shape phenotypic and genotypic similarities among species becoming a challenge in phylogenetic studies.

Pines (genus *Pinus*, family Pinaceae) comprise a group of coniferous tree species occurring almost exclusively in the Northern Hemisphere, with populations often dominant across areas of the North Temperate Zone (Critchfield and Little 1966). They are keystone species of the vast boreal forest ecosystem. They provide pulp and timber products as primary commodities (Lowe *et al.* 1999; Borders and Bailey 2001; Alexander *et al.* 2002; Wear and Greis 2002; Croitoru 2007), but some additional pine forest products may include, for example, pine nuts, berries, herbs and mushrooms (Alexander *et al.* 2002; Bürgi *et al.* 2013; Nagasaka 2013). They provide a pivotal habitat for wildlife species (Brockerhoff *et al.* 2008), and greatly contribute to carbon storage and other ecosystem services (Goodale *et al.* 2002; McKinley *et al.* 2011). Due to their aesthetic value, they add recreational and ornamental dimensions in urban and suburban areas (Tyrväinen and Väänänen 1998; Knoth *et al.* 2002). These qualities extend to southeastern forest ecosystems of the USA, where pines may grow also in mixed forests with hardwoods (Sharitz *et al.* 1992), and where they additionally play an important role in ecosystem recovery from natural disturbances. For example, longleaf pine has developed complex adaptations to fire that allow fast stand regeneration (Outcalt 2000), and loblolly pine helps minimize soil erosion and provides watershed protection due to its fast growth (Schultz 1997).

Four major pines of the southeastern USA were investigated in this study: shortleaf (*Pinus echinata*), slash (*P. elliottii*), longleaf (*P. palustris*) and loblolly (*P. taeda*) (four of the ‘southern pines’; subsection *Australes*, section *Trifoliae*, genus *Pinus*). They are widely cultivated, greatly dominating the southern US forest inventory (Sternitzke and Nelson 1970), and are, therefore, a subject of breeding programmes in the region (Wear and Greis 2002; McKeand *et al.* 2003; Fox *et al.* 2007). The traditional classification (Little and Critchfield 1969) considered 11 species in this subsection with habitat stretching cumulatively from the southeastern USA, through Mexico, to the Caribbean and Central America: slash (*P. elliottii*), spruce (*P. glabra*), longleaf (*P. palustris*), pond (*P. serotina*) and loblolly (*P. taeda*) pines in the southeastern USA; shortleaf (*P. echinata*), Table Mountain (*P. pungens*) and pitch (*P. rigida*) pines in the eastern USA; Cuban pine (*P. cubensis*) in Cuba; West Indian pine (*P. occidentalis*) in the West Indies; and Caribbean pine (*P. caribaea*) in both the West Indies and adjacent Central America. Attempts to refine the phylogeny of *Australes* are ongoing, but typically have been approached in the broader context of the Pinaceae family or along with other subsections.

**Figure 1.**
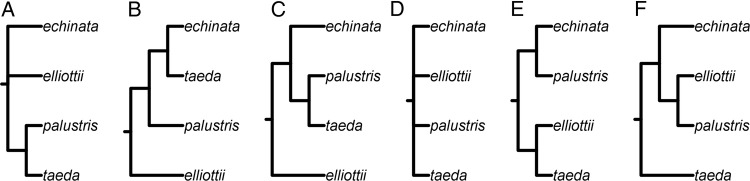
Cladograms for the four *Australes* species—*P. echinata*, *P. elliottii*, *P. palustris* and *P. taeda*—from published studies. (A) Adams and Jackson (1997). (B) Dvorak *et al.* (2000). (C) Grotkopp *et al.* (2004). (D) Gernandt *et al.* (2005). (E) Eckert and Hall (2006). (F) Hernández-León *et al.* (2013). The original studies presented the four species in a broader context along with other pines.

Several previous investigations have provided insights to the phylogenetic relationships of the four species on which we focus in our study (i.e. *P. echinata*, *P. elliottii*, *P. palustris* and *P. taeda*; Fig. 1). Adams and Jackson (1997) found a very close relationship between *P. palustris* and *P. taeda* based on 21 morphological characters, but their relationship to *P. echinata* and *P. elliottii* remained unresolved, and no statistical support was provided. Later, using RAPD markers and the neighbour-joining method, Dvorak *et al.* (2000) suggested a close relationship among *P. echinata*, *P. palustris* and *P. taeda*, in which *P. echinata* and *P. taeda* were sister lineages, and *P. elliottii* was sister to *P. caribaea*. Using a supertree approach and previously published phylogenies based on both morphological and molecular data, Grotkopp *et al.* (2004) also inferred a close relationship among *P. echinata*, *P. palustris* and *P. taeda*, but found that *P. palustris* was sister to *P. taeda*; *P. elliottii* was, again, sister to *P. caribaea*. Gernandt *et al.* (2005) used two chloroplast genes in 101 pine species, but relationships among the four *Australes* species remained unresolved. Eckert and Hall (2006) used four chloroplast genes in 83 pine species. In their study, *P. echinata* and *P. palustris* were placed in one clade with low support, and *P. elliottii* and *P. taeda* were placed in another. Most recently, based on five chloroplast DNA (cpDNA) markers, Hernández-León *et al.* (2013) placed *P. echinata*, *P. elliottii* and *P. palustris* within the same clade, where *P. elliottii* and *P. palustris* were sisters, and *P. taeda* was in a separate clade, albeit with low bootstrap support (BS). Consequently, the monophyly of the four species was placed under question.

Evidence from cpDNA used in recent studies additionally questioned monophyly of the subsection *Australes* as defined by Little and Critchfield (1969), and suggested that the 11 *Australes* species may be scattered throughout a larger clade of over twice as many species (Gernandt *et al.* 2005; Eckert and Hall 2006; Hernández-León *et al.* 2013). Chloroplast genomes, however, may follow different evolutionary trajectories than nuclear genomes, with potentially confounding effects in phylogenetic studies (Rieseberg and Soltis 1991). In pines, chloroplast genomes are strictly paternally inherited, thus having smaller effective population sizes than nuclear genomes. Additionally, cpDNA experiences lower substitution rates (Willyard *et al.* 2007). Consequently, these factors may lead to discordant patterns of polymorphism between the two genomes (Soltis *et al.* 1992; Hong *et al.* 1993*b*). Moreover, cpDNA differentiation among populations of a species can be high (Hong *et al.* 1993*a*), and the cpDNA sequence variation in pines may be unevenly distributed throughout the genome requiring a representative locus sampling (Whittall *et al.* 2010). Finally, foreign chloroplast genomes introduced through hybridization may undergo a rapid fixation—chloroplast capture (Rieseberg and Soltis 1991; Matos and Schaal 2000; Liston *et al.* 2007).

Apart from the specificity of cpDNA evolution, additional problems with taxonomic classification of *Australes* may stem from incomplete lineage sorting, a phenomenon reported in pines (Syring *et al.* 2007; Willyard *et al.* 2009), especially considering the relatively recent evolutionary history of the subsection. Radiation within *Australes* is thought to have begun only 7–15 million years ago (MYA) (Willyard *et al.* 2007; Hernández-León *et al.* 2013). Large overlap in present-day areas of the four species studied here has fostered occasional or historic hybridization between some of them that may have additionally contributed to insufficient resolution in phylogenetic studies. Natural hybridization has been observed between *P. echinata* and *P. taeda* (Zobel 1953; Mergen *et al.* 1965; Smouse and Saylor 1973; Edwards-Burke *et al.* 1997), between *P. palustris* and *P. taeda* (Chapman 1922) and between *P. elliottii* and *P. palustris* (Mergen 1958). Almost all possible hybrids for these species can be artificially generated in controlled crosses, with the exception of *P. echinata* and *P. palustris* that are not considered interfertile despite a reported hybrid case (Snyder and Squillace 1966; Campbell *et al.* 1969). Additionally, similar environmental constraints could lead to a parallel support for beneficial alleles and/or removal of detrimental ones. Nevertheless, sympatry could have been repeatedly disturbed in the past; for example, during the Pleistocene, *P. taeda* was likely constricted to two refugia, *P. elliottii* and *P. palustris* could have been separated, each one occupying one of the two refugia, while *P. echinata*'s range was probably continuous due to its cold-hardiness (Wells *et al.* 1991; Schmidtling and Hipkins 1998; Schmidtling 2003). Each of these factors, or their combination, could confound phylogenetic inference.

The challenge with inferring relationships among *Australes* indicates that more work is needed. Nuclear markers are recommended to use when introgression, cytoplasmic in particular, or lineage sorting phenomena are present (Soltis *et al.* 1992). Consequently, we used 11 nuclear protein coding genes. Our objective was to refine and potentially clarify the phylogenetic relationships among *P. echinata*, *P. elliottii*, *P. palustris* and *P. taeda. Pinus pinaster* was used as an outgroup. Despite the larger dataset and application of advanced methods, untangling their phylogeny was not straightforward. We discuss potential factors that likely contributed to this problem and could explain the difficulties in inferring phylogenies using multiple genes observed in previous studies.

## Methods

### Source of data

Data from 32 genes recently sequenced and annotated in four pines from subsection *Australes*, namely *P. taeda* (Brown *et al.* 2004; González-Martínez *et al.* 2006), *P. echinata*, *P. elliottii* and *P. palustris* (Koralewski *et al.* 2014), were used to query the NCBI GenBank database (Benson *et al.* 2013) for orthologs in related pine (genus *Pinus*) taxa that could be used as appropriate outgroup species. Eleven putative orthologous genes were identified in *P. pinaster* (Table 1), which was used as an outgroup. Given that the ingroup species belong to a single subsection, it is possible that a species from a more closely related subsection could be a more optimal outgroup for phylogenetic analyses; however, *P. pinaster* is the best studied species with respect to the genes investigated in the southern pines, allowing us to utilize more sequence data in the analyses. We assumed orthology of the genes based on exon–intron structure and very high sequence similarity. For each putative ortholog, the *E*-value was 3E-46 or less and identity score was 95 % or more in at least one comparison of an ingroup species with *P. pinaster*; additionally, in comparisons where the *E*-value was higher than E-100, the identity score was 99 %. However, well-annotated, mapped and well-assembled whole-genome data are ultimately needed to validate orthology. Such data are still unavailable, although the first incomplete draft genome assembly was published recently for loblolly pine (Neale *et al.* 2014; Zimin *et al.* 2014).

**Table 1.** Genes used in the study and their NCBI accession numbers. Genomic DNA sequences for the ingroup (*P. echinata*, *P. elliottii*, *P. palustris* and *P. taeda*) were newly generated and presented in Koralewski *et al.* (2014), and both genomic DNA and mRNA sequences for the outgroup (*P. pinaster*) were already available in NCBI.

Gene name	Gene abbreviation	*Pinus echinata*	*Pinus elliottii*	*Pinus palustris*	*Pinus taeda*	*Pinus pinaster*
*4-coumarate:CoA ligase*	*4cl*	KF158811	KF158813	KF158814	KF158816	HM482497
*arabinogalactan 4*	*agp-4*	KF158819	KF158821	KF158822	KF158824	AM501931
*trans-cinnamate 4-hydroxylase 2*	*c4h-2*	KF158875	KF158877	KF158878	KF158880	JN013973
*cinnamyl alcohol dehydrogenase*	*cad*	KF158882	KF158883	KF158884	KF158885	FN824799
*cellulose synthase*	*cesA3*	KF158898	KF158900	KF158901	KF158903	FN257074
*caffeate O-methyltransferase*	*comt-2*	KF158906	KF158908	KF158909	KF158911	HE574557
*dehydrin 2*	*dhn-2*	KF158924	KF158926	KF158927	KF158929	HE796687
*early response to drought 3*	*erd3*	KF158931	KF158932	KF158933	KF158934	EU020011
*glycine hydroxymethyltransferase*	*glyhmt*	KF158935	KF158936	KF158937	KF158938	HE574564
*ABII protein phosphatase 2C-like*	*pp2c*	KF158952	KF158953	KF158954	KF158955	EU020014
*chloroplast Cu/Zn superoxide dismutase*	*sod-chl*	KF158978	KF158979	KF158980	KF158981	AF434186

### Multiple alignment

The FASTA sequences for individual genes were aligned using BioEdit (ver. 7.0.9.0) (Hall 1999) and merged into one dataset in SeaView (ver. 4.0) (Galtier *et al.* 1996). Conversion from FASTA to NEXUS was done in SeaView, and from FASTA to PHYLIP manually in the text editor Notepad++ (ver. 5.9.3) (Ho 2011). Coding sites were assigned based on annotation data in NCBI GenBank for each individual gene separately using DnaSP (ver. 5.10.01) (Librado and Rozas 2009). The merged NEXUS file was further manually annotated, and one of five categories was assigned to each site: a codon position (1, 2 or 3), intron or 3′UTR. All sites with missing data were removed from the analysis **[see Supporting Information—Dataset S1]**.

### Partitioning

Partitioning schemes and models of molecular evolution were evaluated using PartitionFinder (ver. 1.1.1) (Lanfear *et al.* 2012). Model selection was restricted to the set of models implemented in the software in which we intended to run the phylogenetic analysis (parameter ‘model =’ in PartitionFinder). At most 42 models were evaluated simultaneously based on Akaike information criterion (AIC) and Bayesian information criterion (BIC). The parameters describing gamma distribution of rates among sites (Γ or G) and a proportion of invariable sites (I) were considered among the models exclusively, i.e. none of the evaluated models accounted for both Γ and I jointly, because of reported problems with independent optimization of these two parameters (see discussions and relevant references in Stamatakis 2008: pp. 20–21 and in Stamatakis 2014: p. 49). Additionally, in the group of models evaluated for GARLI (Zwickl 2006) (see below), one or more of the K81 models (K81, K81 + I and K81 + G, depending on the subset of data; a few data subsets were affected) caused convergence problems and were not considered. Depending on the intended phylogenetic analysis, alternative partitioning schemes were then implemented **[see Supporting Information—Table S1]**: (i) by-gene-site: best partitioning schemes for 39 sets corresponding to different genes and site categories within genes, or (ii) by-gene: best models identified for each of the 11 genes in the set (no partitioning within a gene). Additionally, in the cases where each gene was analysed separately, models were identified for every gene independently with sites assigned to one of the five site categories (by-site).

### Phylogenetic analysis

The combined dataset of 11 genes was subjected to two gene tree methods with partitioning by-gene-site: maximum likelihood (ML; GARLI, ver. 2.01) (Zwickl 2006) and Bayesian inference (BI; MrBayes, ver. 3.2.2) (Ronquist *et al.* 2012). Species trees were reconstructed using the Bayesian method BEST (ver. 2.3) (Liu 2008) with partitioning by-gene. To account for potential polytomies, and thus potential for the star tree paradox (Suzuki *et al.* 2002; Lewis *et al.* 2005), we ran Phycas (ver. 1.2.0) (Lewis *et al.* 2010), also a Bayesian method, with partitioning by-gene-site. We then analysed each gene separately using GARLI and MrBayes (partitioning by-site). The MrBayes outputs were further investigated using Bayesian concordance analysis (Ané *et al.* 2007), as implemented in BUCKy (ver. 1.4.0, mbsum ver. 1.4.2) (Larget *et al.* 2010), for seven values of *α*: 0.01, 0.5, 1, 2, 5, 10 and 10 000. The value of *α* corresponds to the probability that loci share the same tree (the lower *α* the higher the probability, and vice versa), thus affecting the clade support (the higher the probability the higher support). Formally, BUCKy is not considered a species-tree method; however, the resulting primary concordance tree can be generally considered comparable with species trees (for an in-depth discussion about the species methods, see Mateos *et al.* 2012).

All analyses were run in Windows 7 **[see Supporting Information—Table S1]** except for BUCKy, which was compiled and run under Cygwin. The tree figures were visualized in FigTree (ver. 1.4) (Rambaut 2013) and further edited manually to increase their readability and compactness.

### Clade support and convergence

In order to determine the clade support, BS was calculated in GARLI using 1000 replicates **[see Supporting Information—Table S1]**. SumTrees (ver. 3.3.1) (Sukumaran and Holder 2010) was used to generate majority consensus trees. Posterior probability (PP) estimates were used for the BI methods. To verify that the Markov chain Monte Carlo analysis converged on a stationary distribution and that the sampling of the distribution was adequate, the following criteria were applied for MrBayes and BEST: (i) stable PP values, (ii) small and stable average standard deviation of the split frequencies of independent runs, (iii) potential scale reduction factor close to 1 and (iv) an effective sample size of at least 200 for the posterior probabilities. The conditions (i) and (iv) were evaluated also for Phycas, and additionally the split PP plot and split sojourn plot were examined. Samples prior to reaching stationarity were discarded as ‘burnin’. The conditions (i) and (iv) were evaluated in Tracer (ver. 1.5.0) (Rambaut and Drummond 2009). Average standard deviation of mean sample-wide concordance factor (CF) was examined for BUCKy, and the CF was used to determine clade support.

## Results

### Combined evidence from all genes

**Figure 2.**
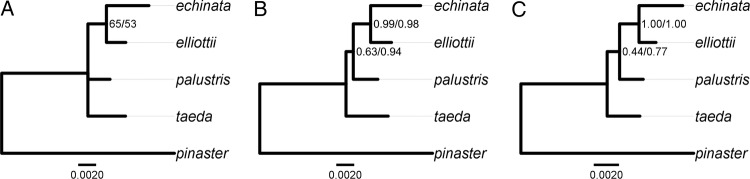
Joint analysis of 11 genes in *P. echinata*, *P. elliottii*, *P. palustris* and *P. taeda* using GARLI (A), MrBayes (B) and Phycas (C). Numbers at nodes correspond to BS for GARLI (%; AIC/BIC), and PP for MrBayes and Phycas (AIC/BIC). Branch lengths are shown for the BIC partitioning schemes.

Three methods, GARLI, MrBayes and Phycas, recovered the clade *echinata*–*elliottii* for the concatenated matrix of 11 genes. Support from the ML method was much lower (highest BS = 65 %, AIC partitioning) than that from the BI methods (lowest PP = 0.98 in MrBayes, BIC partitioning). The BI methods additionally supported the clade *echinata*–*elliottii*–*palustris*, but support varied considerably (from PP < 0.50 in Phycas, AIC, to PP = 0.94 in MrBayes, BIC). Significant differences between the ML and BI methods in support for the clade *echinata*–*elliottii*, and in the case of the clade *echinata*–*elliottii*–*palustris*, also between Phycas and MrBayes (Fig. 2) are characteristic signatures of true or approximate star phylogenies (Suzuki *et al.* 2002; Lewis *et al.* 2005).

**Figure 3.**
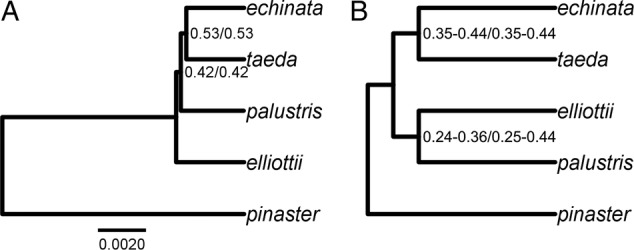
Joint analysis of 11 genes in *P. echinata*, *P. elliottii*, *P. palustris* and *P. taeda* using BEST (A) and BUCKy (cladogram; B). Numbers at nodes correspond to PP for BEST (AIC/BIC) and CFs for BUCKy (AIC/BIC; range of CFs for *α* values from 10 000 to 0.01).

BEST supported the clade *echinata*–*taeda*, absent in the gene-tree methods, with PP = 0.53 (AIC and BIC; Fig. 3). This clade was present also in the primary concordance tree in BUCKy (CF ranging from 0.35 to 0.44, depending on *α* and model selection criterion). Another clade present in the BUCKy's primary concordance tree was *elliottii*–*palustris* (CF ranging from 0.24 to 0.44, depending on *α* and model selection criterion).

### Individual gene approach

**Figure 4.**
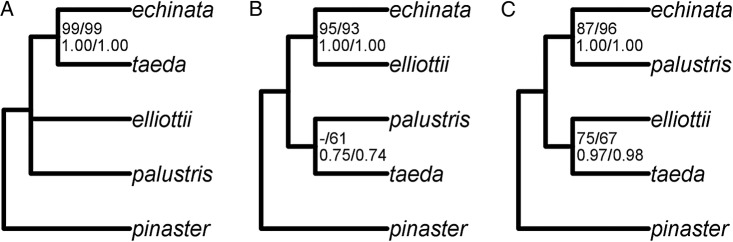
Analysis of three groups of genes: Group A (*4cl*, *c4h-2* and *cesA3*; A), Group B (*agp-4* and *sod-chl*; B) and Group C (*dhn-2* and *erd3*; C) in *P. echinata*, *P. elliottii*, *P. palustris* and *P. taeda* using GARLI and MrBayes. Cladograms are shown. Numbers at nodes correspond to clade support: GARLI (AIC/BIC; top row) and MrBayes (AIC/BIC; bottom row). The BS for the clade *palustris*–*taeda* in Group B was <0.50.

Given the results from the analysis of the combined dataset resulting in mostly unresolved relationships among the studied species, and the conflicts found between the gene-tree and species-tree methods, we looked at each gene separately using ML (GARLI) and BI (MrBayes). The two approaches produced consistent results for each gene, although the difference in clade support varied among the genes **[see Supporting Information—Fig. S1]**. We, therefore, clustered genes into three groups following similarity in topologies. The genes *4cl*, *c4h-2* and *cesA3* (Group A) supported the clade *echinata*–*taeda*, the genes *agp-4* and *sod-chl* (Group B) supported the clade *echinata*–*elliottii* and the genes *dhn-2* and *erd3* (Group C) supported the clade *echinata*–*palustris*. Group C genes disagreed in the placement of the species *P. elliottii* and *P. taeda*; however, we decided to focus on the similarity. Additional analysis in the R environment (ver. 2.15.2) (R Core Team 2012) supported these groupings—principal coordinate analysis (function pcoa, package ape ver. 3.0-11) (Paradis *et al.* 2004) run on Robinson–Foulds distance matrix for the individual gene trees (function RF.dist, package phangorn ver. 1.99-7) (Schliep 2011) placed the three groups of genes in distinct clusters **[see Supporting Information—Fig. S2]**. Each set was partitioned by-gene-site, and analysed using GARLI and MrBayes. In general, the BS for the clades jointly supported by genes within each group increased, the PP reached (or stayed at) 1.00 and the level of support for these clades became consistent between GARLI and MrBayes (Fig. 4) **[see Supporting Information—Fig. S3]**. In the case of Group B, the clade *palustris*–*taeda*, previously present only in the *agp-4* gene tree, was also recovered, although with low support. In Group C, *P. elliottii* and *P. taeda* were placed as sister taxa in a clade with low BS and high PP, which reflects the conflict between the two individual gene trees.

## Discussion

The discordance among various approaches applied in this study mirrors the conflicting results from previously published work (see Introduction). Comparison of the topologies supported by Group A vs. Group B vs. Group C genes shows highly consistent results within each group across different methods, yet the conclusions contradict each other among the groups. The clade *echinata*−*taeda*, supported by Group A genes, was previously recovered by Dvorak *et al.* (2000) using RAPD markers and the neighbour-joining method with BS = 0.90. The species in the clade *palustris*–*taeda*, supported by the gene *agp-4* (Group B), were placed in one clade by Adams and Jackson (1997) based on parsimony analysis of morphological characters, and by Grotkopp *et al.* (2004) through a supertree approach (57 % of individual trees agreed with the supertree at the node). Both clades *echinata*–*palustris* and *elliottii*–*taeda* supported by the Group C genes corresponded to grouping in Eckert and Hall (2006), who used chloroplast data.

Given life history of the subsection (see Introduction), long-distance gene flow through pollen, hybridization events that still occur today and large (but variable in the past) population sizes of the investigated species, incomplete lineage sorting could be the simplest and most straightforward explanation of the pattern observed in our study. It is a known phenomenon in pines (Syring *et al.* 2007; Willyard *et al.* 2009), and it is likely to confound phylogenetic inference in a clade as recent as *Australes*. Interestingly, however, the genes forming the three groups are also connected at the putative functional level (Table 2). The Group A genes all directly affect wood properties: *4cl* and *c4h-2* are both part of the lignin biosynthesis pathway (Whetten and Sederoff 1995; Boerjan *et al.* 2003), and *cesA3* is involved in cellulose synthesis (Pot *et al.* 2005). Another gene in our dataset, *comt-2*, is also involved in lignin biosynthesis (Whetten and Sederoff 1995; Boerjan *et al.* 2003) but did not recover the *echinata*–*taeda* clade. Unlike the other three genes in Group A, it supported the clade *elliottii*–*palustris*. An additional joint analysis of all four genes, using the same methodology, showed that the support for the previously recovered clade *echinata*–*taeda* almost did not change, while the clade *elliottii*–*palustris* received a low-to-moderate support **[see Supporting Information—Fig. S4]**. The latter two species were previously placed in one clade (BS ≤ 0.50) by Hernández-León *et al.* (2013) based on an ML method applied to plastid data. Conversely, the Group B and C genes are not involved in the lignin biosynthesis pathway directly. The Group B genes (*agp-4* and *sod-chl*) appear to contribute to plant defence, water management and other functions. Proteins from the arabinogalactan family play signalling and protective roles, and participate in xylem development, cell growth and expansion, programmed cell death and other processes (Loopstra and Sederoff 1995; Loopstra *et al.* 2000; Zhang *et al.* 2000, 2003; Showalter 2001; Seifert and Roberts 2007). *Sod-chl* has antioxidative properties (Huttunen and Heiska 1988; Bowler *et al.* 1992). It was also drought responsive (Costa *et al.* 1998) and hence was considered a drought-tolerance candidate gene in loblolly pine (González-Martínez *et al.* 2006). *Dhn-2* and *erd3* (Group C) are also associated with water stress recognition and response. *Dhn-2*, a *dehydrin*, is responsive to dehydration stress and also plays other protective functions (González-Martínez *et al.* 2006; Eveno *et al.* 2008). The role of *erd3* is less known, but it has been found active in an early stage of dehydration stress (González-Martínez *et al.* 2006; Eveno *et al.* 2008).

**Table 2.** Groups of genes based on phylogenetic analysis of *P. echinata*, *P. elliottii*, *P. palustris* and *P. taeda*, their selected putative functions and numbers of nonsynonymous (N) and silent (S) nucleotide substitutions in each group.

Group	Genes	Putative function	N	S
A	*4cl*, *c4h-2* and *cesA3*	Wood properties	2	10
B	*agp-4* and *sod-chl*	Plant defence, water management	4	10
C	*dhn-2* and *erd3*	Water stress recognition and response	2	11

Regardless of the extent of the potential effects of shared ancestral polymorphisms, two other hypotheses of parallel evolution within the functional gene groups could be pursued in follow-up studies. The functionally bound clades could have resulted from a transfer of adaptive alleles via introgressive hybridization from an adapted pine donor, followed by positive selection and subsequent purifying or balancing selection acting in both donor and acceptor populations. Alternatively, speciation could have begun while the locus was already under purifying or balancing selection, which continued working simultaneously in both populations in parallel, given common habitat locations and environmental pressures. Sympatry, shifting locale of the optimum habitat, population size changes and the recent evolutionary origin of the southern pines could have facilitated hybridization, while crucial roles of the genes belonging to the three groups could have resulted in preservation of adaptive variants, especially under similar environmental selection pressures. A tight physical linkage among the loci within each group can be excluded as an alternative possible explanation for the observed phenomenon. Most of the genes studied here are mapped to different linkage groups in *P. taeda*, or far from each other, although no linkage data were found for *dhn-2* and *erd3* **[see Supporting Information—Table S2]**. These two hypotheses require more assumptions to explain the observed pattern when compared with the hypothesis of incomplete lineage sorting, and therefore, the latter should be preferred, although some form of interplay of all three is certainly possible.

In order to reject the hypothesis of incomplete lineage sorting, and to pursue an alternative, limitations of our study need to be overcome. Multiple individuals sampled for each species would allow for thorough identification of shared ancestral polymorphisms and variation fixed at the species level. Including more genes per functional group would allow to examine whether the observed pattern holds also for other members of a given pathway or process, or if it is purely stochastic. Samples from all members of the clade *Australes* would likely help improve overall robustness of the phylogenetic inference. Additionally, to test the monophyly of the clade *Australes* in the traditional sense (Little and Critchfield 1969), which has been questioned by cpDNA-based studies (Gernandt *et al.* 2005; Eckert and Hall 2006; Hernández-León *et al.* 2013), species traditionally classified as *Attenuatae*, *Leiophyllae* and *Oocarpae* should be included in the nuclear marker-based study. Alternative outgroup species could also be considered, especially those from subsections *Contortae* and *Ponderosae* that are more closely related to the ingroup than *P. pinaster*. Solving these caveats, however, requires additional resources and cannot be done purely analytically. Given the limitations of the data, our primary intention was to apply a spectrum of methods to a gene sample that would maximize genome coverage.

Recent estimates of radiation within *Australes* suggested that it could have begun as recently as 7 MYA (*P. taeda*–*P. radiata* split), although the split between the ancestors of *Ponderosae* and *Australes* might have happened as early as 15 MYA (Willyard *et al.* 2007). This timeframe overlaps with the mid- to late-Miocene (about 14–15 MYA), starting at or directly following the middle Miocene climate transition (MMCT), a period of cooling and ice-sheet expansion that took place about 14 MYA (Shevenell *et al.* 2004). Pines experienced habitat locale shifts both before and after the MMCT, for example, during the Eocene (56–34 MYA), interpreted as the major stimulus for pine divergence at the time, and during the Pleistocene (2.6–0.01 MYA) (Millar 1993). The MMCT likely affected population sizes, species range and distribution of the allelic variation, and probably had the momentum to trigger radiation within *Australes*. The potential for hybridization events resulting in introgression was likely greater back in time, especially when the ancestral species were far less diverged and stressed by recurrent changes in environmental pressures and by range shifts. Range expansions could have then brought multiple genetic effects (Excoffier *et al.* 2009) including increase in frequency of rare (and also newly introduced) variants. This process would have happened much faster if the newly acquired alleles were advantageous.

The adaptations shared among the southern pines and shaped by the vibrant historic climate are particularly interesting in the light of the ongoing and forthcoming climate changes, amidst the discussion on assisted migration (Vitt *et al.* 2010; Krutovsky *et al.* 2012; Koralewski *et al.* 2015). The historical events might have led to increased standing genetic variation in these species, directly influencing their level of adaptability and making them somewhat ‘climate-change ready’. Additional inquiries directed towards the loci studied here could improve breeding strategies in the face of climate change (Krutovsky *et al.* 2013).

## Conclusions

Incomplete lineage sorting, introgression and parallel evolution can explain inconsistencies observed in the phylogenetic analysis of the four southern pines. However, more data are needed to discriminate among these hypotheses. The conflicting signals were vigorously tested, but evidence in the current data was not robust enough to support potent claims, and thus, the simplest hypothesis of incomplete lineage sorting may be preferred, while the alternatives may be pursued in future studies. To overcome limitations of our study, additional sampling should include multiple individuals per species, additional species that form one clade with the four pines investigated here, less distant outgroup species and additional functionally related genes. Our work provided new insights into the *Australes* phylogeny, but their evolutionary history remains elusive.

## Sources of Funding

The project was supported by the United States Department of Agriculture (USDA) Cooperative State Research, Education, and Extension Service (CSREES) and Texas Agricultural Experiment Station (TAES) McIntire-Stennis Project (TEX09122-0210381). The Pine Integrated Network: Education, Mitigation, and Adaptation Project (PINEMAP), a Coordinated Agricultural Project funded by the USDA National Institute of Food and Agriculture, Award #2011-68002-30185, provided support during preparation of this manuscript.

## Contributions by the Authors

T.E.K., M.M. and K.V.K. conceived and designed the study. T.E.K. arranged the data and ran the software. T.E.K., M.M. and K.V.K. interpreted results. T.E.K. drafted the manuscript. T.E.K., M.M. and K.V.K. edited, read and approved the final version of the manuscript.

## Conflict of Interest Statement

None declared.

## Supporting Information

The following additional information is available in the online version of this article —

**Dataset S1.** Multiple alignment in NEXUS format.

**Figure S1.** Separate analysis of each gene for the four *Australes* species: *P. echinata*, *P. elliottii*, *P. palustris* and *P. taeda*. Cladograms are shown. From top to bottom: *4cl*, *c4h-2* and *cesA3* (A); *agp-4* and *sod-chl* (B); *dhn-2* and *erd3* (C); *cad*, *glyhmt*, *pp2c* and *comt-2* (D). Numbers at nodes correspond to clade support: GARLI (AIC/BIC; top row) and MrBayes (AIC/BIC; bottom row).

**Figure S2.** Principal coordinate analysis run on Robinson–Foulds distance matrix for the individual gene trees. Group A gene names (*4cl*, *c4h-2* and *cesA3*) are in green, Group B gene names (*agp-4* and *sod-chl*) are in orange and Group C gene names (*dhn-2* and *erd3*) are in blue.

**Figure S3.** Joint analysis of the genes from Group A (*4cl*, *c4h-2* and *cesA3*; A), Group B (*agp-4* and *sod-chl*; B) and Group C (*dhn-2* and *erd3*; C) in *P. echinata*, *P. elliottii*, *P. palustris* and *P. taeda* using GARLI and MrBayes. From top to bottom: GARLI (AIC), GARLI (BIC), MrBayes (AIC) and MrBayes (BIC). Numbers at nodes correspond to clade support.

**Figure S4.** Joint analysis of the genes from Group A (*4cl*, *c4h-2* and *cesA3*) and *comt-2* in *P. echinata*, *P. elliottii*, *P. palustris* and *P. taeda* using GARLI and MrBayes. From top to bottom: GARLI (AIC), GARLI (BIC), MrBayes (AIC) and MrBayes (BIC). Numbers at nodes correspond to clade support.

**Table S1.** Partitioning schemes and software settings.

**Table S2.** Linkage information for the studied genes.

Additional Information
