# Supplementary material for: Conflicting genomic signals affect phylogenetic inference in four species of North American pines
Source: AoB Plants. 2016 Apr 8;8:plw019. doi: 10.1093/aobpla/plw019 (PMC4866652; doi:10.1093/aobpla/plw019)
Supplement: Additional Information [file supp_8_plw019_index.html]

Conflicting genomic signals affect phylogenetic inference in four species of North American pines — Additional Information 

# Conflicting genomic signals affect phylogenetic inference in four species of North American pines

## Additional Information

Additional Information

- Supplementary data\_files1 - doc file
- Supplementary data\_files2 - txt file
- Supplementary data\_files3 - xlsx file
- Supplementary data\_files4 - doc file
