# Supplementary material for: Conflicting genomic signals affect phylogenetic inference in four species of North American pines
Source: AoB Plants. 2016 Apr 8;8:plw019. doi: 10.1093/aobpla/plw019 (PMC4866652; doi:10.1093/aobpla/plw019)
Supplement: Additional Information [file supp_plw019_plw019supp_data_files1.doc]

Article title: Conflicting genomic signals affect phylogenetic inference in four species of North American pines

Supporting Information – Figures S1, S2, S3 and S4


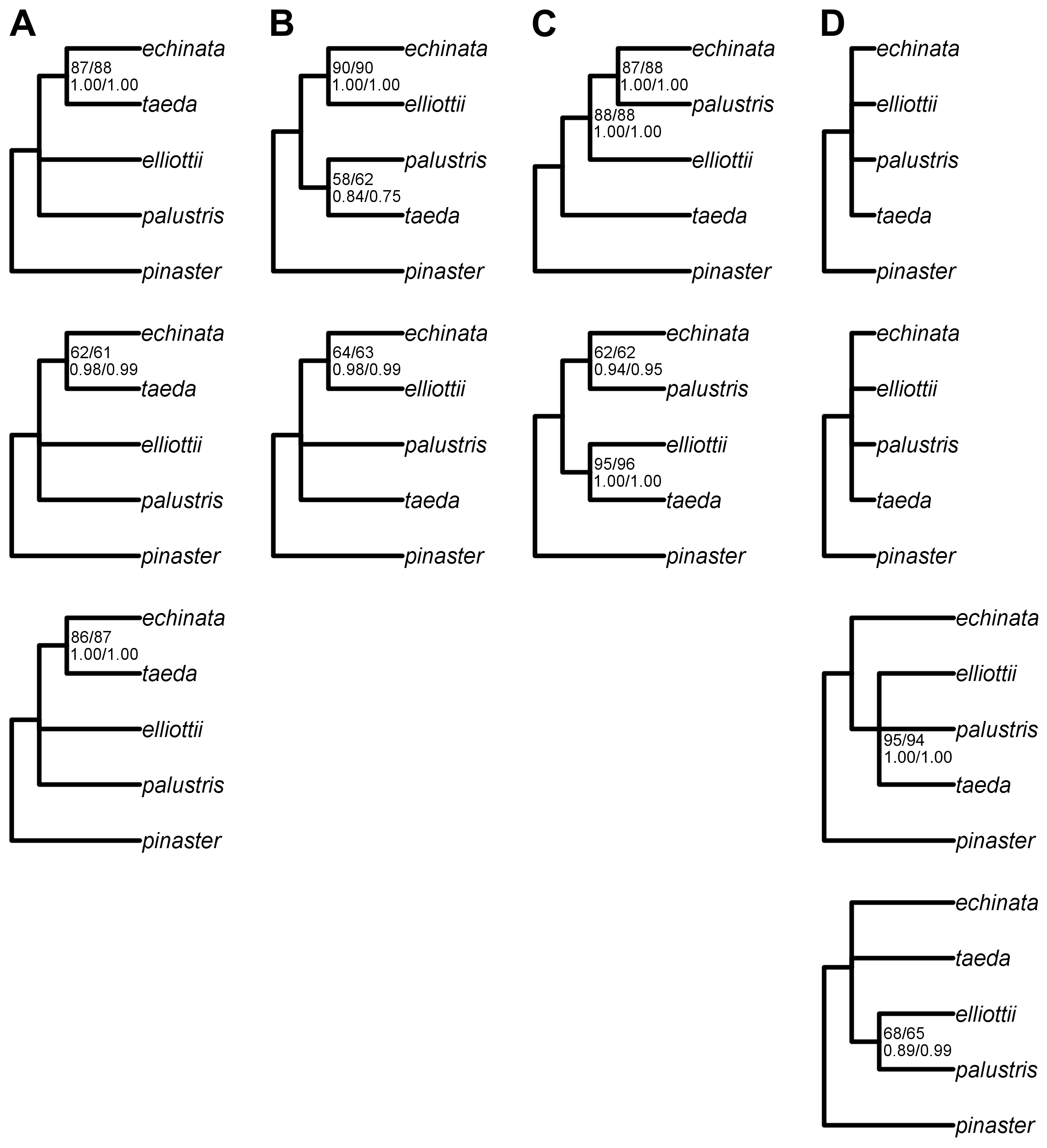


**Figure S1.** Separate analysis of each gene for the four *Australes* species: *P. echinata*, *P. elliottii*, *P. palustris* and *P. taeda*. Cladograms are shown. From top to bottom: *4cl*, *c4h-2* and *cesA3* (A); *agp-4* and *sod-chl* (B); *dhn-2* and *erd3* (C); *cad*, *glyhmt*, *pp2c* and *comt-2* (D). Numbers at nodes correspond to clade support: GARLI (AIC/BIC; top row) and MrBayes (AIC/BIC; bottom row).


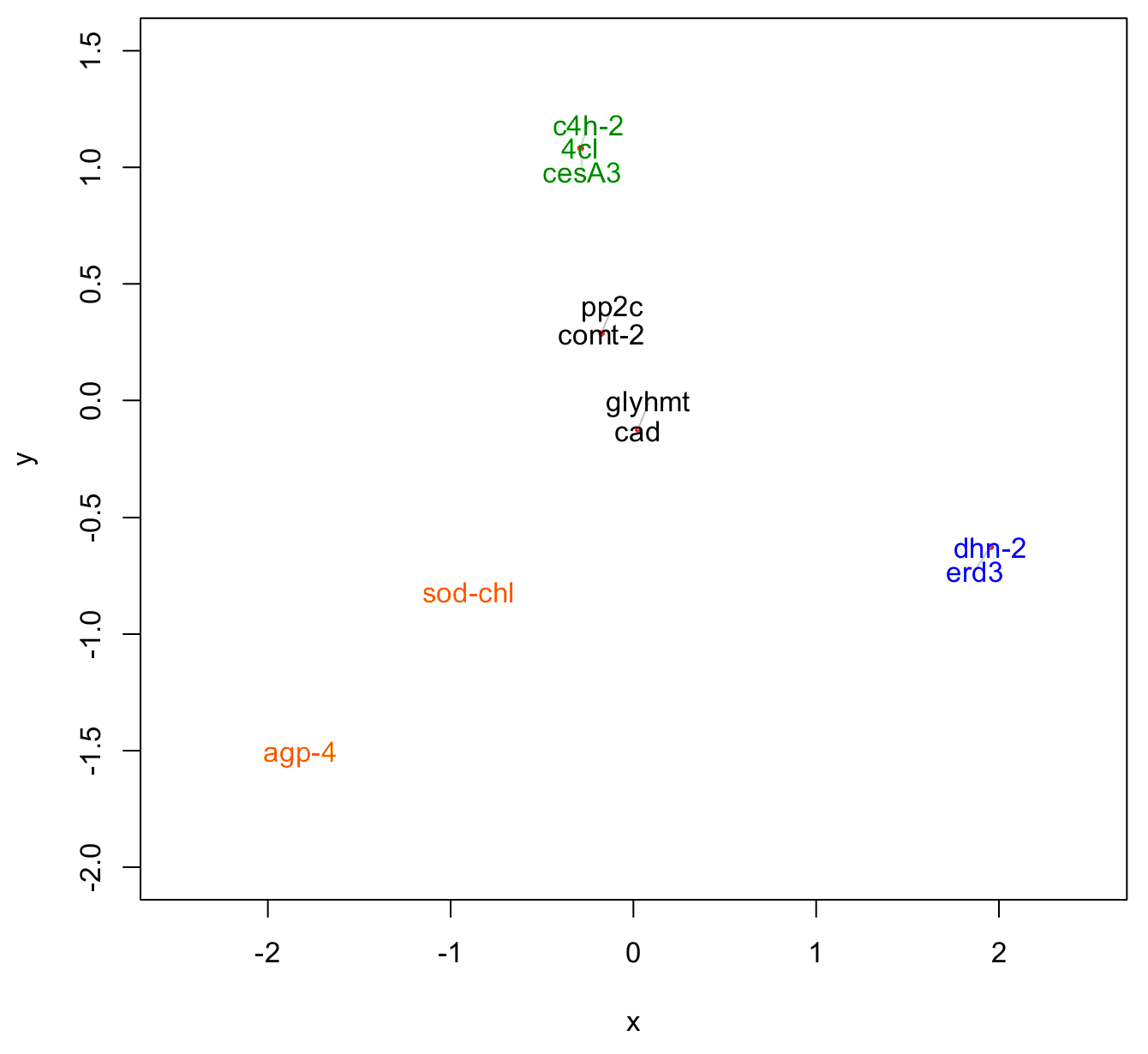


**Figure S2.** Principal coordinate analysis run on Robinson–Foulds distance matrix for the individual gene trees. Group A gene names (*4cl*, *c4h-2* and *cesA3*) are in green, Group B gene names (*agp-4* and *sod-chl*) are in orange and Group C gene names (*dhn-2* and *erd3*) are in blue.


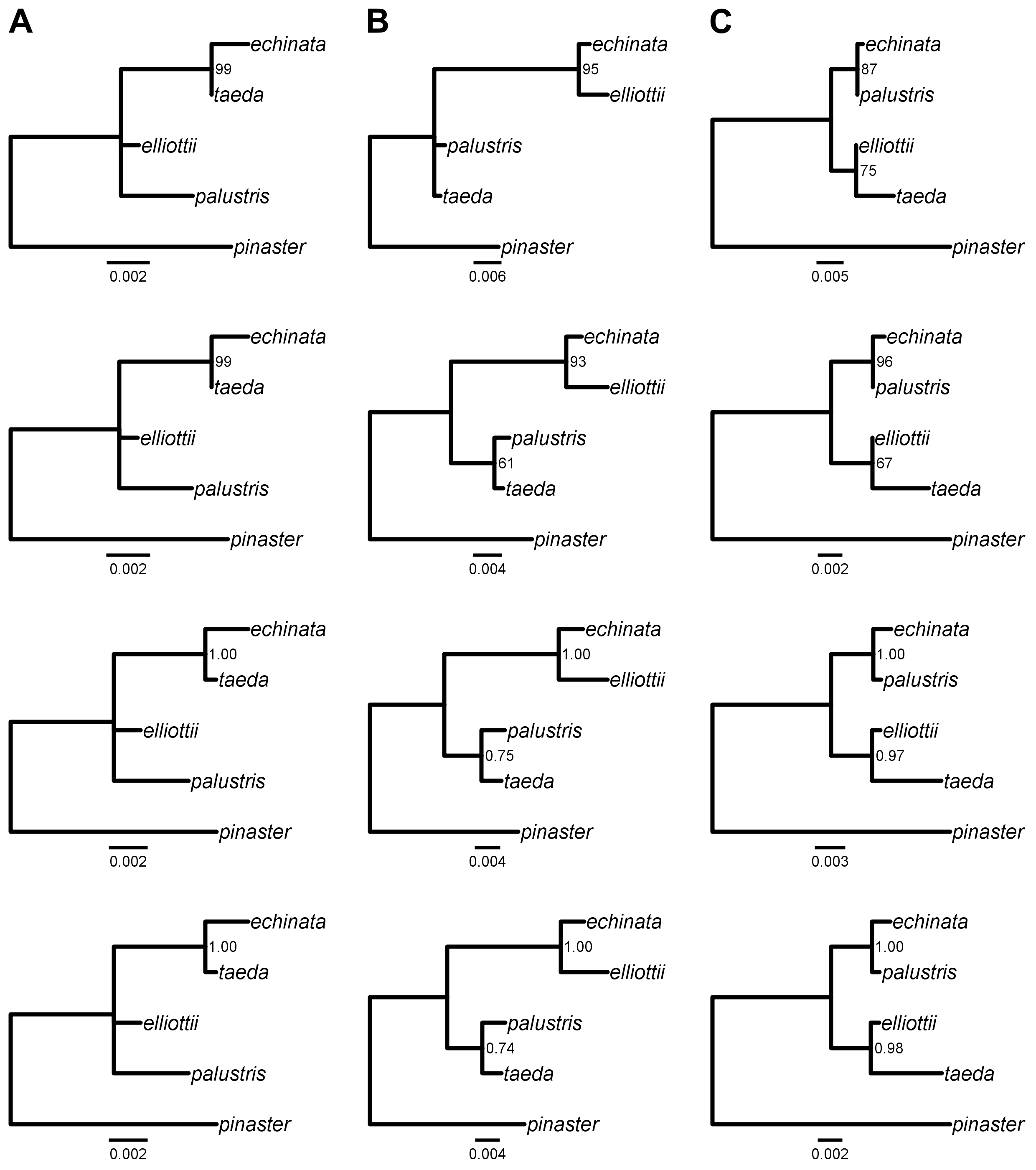


**Figure S3.** Joint analysis of the genes from Group A (*4cl*, *c4h-2* and *cesA3*; A), Group B (*agp-4* and *sod-chl*; B) and Group C (*dhn-2* and *erd3*; C) in *P. echinata*, *P. elliottii*, *P. palustris* and *P. taeda* using GARLI and MrBayes. From top to bottom: GARLI (AIC), GARLI (BIC), MrBayes (AIC) and MrBayes (BIC). Numbers at nodes correspond to clade support.


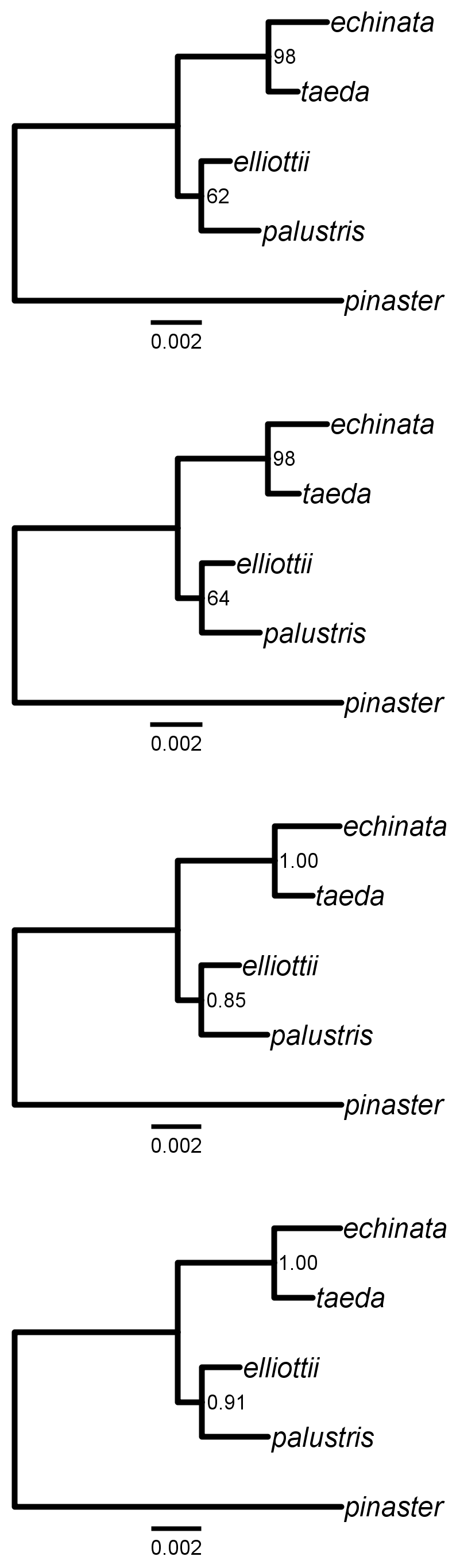


**Figure S4.** Joint analysis of the genes from Group A (*4cl*, *c4h-2* and *cesA3*) and
*comt-2* in *P. echinata*, *P. elliottii*, *P. palustris* and *P. taeda* using GARLI and MrBayes. From top to bottom: GARLI (AIC), GARLI (BIC), MrBayes (AIC) and MrBayes (BIC). Numbers at nodes correspond to clade support.
