# Supplementary material for: Conflicting genomic signals affect phylogenetic inference in four species of North American pines
Source: AoB Plants. 2016 Apr 8;8:plw019. doi: 10.1093/aobpla/plw019 (PMC4866652; doi:10.1093/aobpla/plw019)
Supplement: Additional Information [file supp_plw019_plw019supp_data_files4.doc]

Article title: Conflicting genomic signals affect phylogenetic inference in four species of North American pines

Supporting Information – Table S2

**Table S2. Linkage information for the studied genes.**

| **Gene** | **Group** | **LG** | **References** |
| --- | --- | --- | --- |
| *4cl* | A | 7 | 1, 2, 3, 4 |
| *c4h-2* | A | 10 | 3 |
| *cesA3* | A | 11 | 1 |
| *comt-2* | A extended | 11 | 1, 2, 3, 4 |
| *agp-4* | B | 7 | 1, 2 |
| *sod-chl* | B | 10 | 3, 4 |
| *dhn-2* | C |  |  |
| *erd3* | C |  |  |
| *glyhmt* | - | 3 | 1, 2 |
| *pp2c* | - | 10 | 4, 5 |
| *cad* | - | 9 | 1, 2, 3, 4 |

1. Brown GR, Gill GP, Kuntz RJ, Langley CH, Neale DB. 2004. Nucleotide diversity and linkage disequilibrium in loblolly pine. *Proceedings of the National Academy of Sciences of the United States of America* **101**:15255-15260.

2. Brown GR, Bassoni DL, Gill GP, Fontana JR, Wheeler NC, Megraw RA, Davis MF, Sewell MM, Tuskan GA, Neale DB. 2003. Identification of quantitative trait loci influencing wood property traits in loblolly pine (*Pinus taeda* L.). III. QTL verification and candidate gene mapping. *Genetics* **164**:1537-1546.

3. Echt CS, Saha S, Krutovsky KV, Wimalanathan K, Erpelding JE, Liang C, Nelson CD. 2011. An annotated genetic map of loblolly pine based on microsatellite and cDNA markers. *BMC Genetics* **12**:17.

4. Eckert AJ, Pande B, Ersoz ES, Wright MH, Rashbrook VK, Nicolet CM, Neale DB. 2009. High-throughput genotyping and mapping of single nucleotide polymorphisms in loblolly pine (*Pinus taeda* L.). *Tree Genetics & Genomes* **5**:225-234.

5. González-Martínez SC, Ersoz E, Brown GR, Wheeler NC, Neale DB. 2006. DNA sequence variation and selection of tag single-nucleotide polymorphisms at candidate genes for drought-stress response in *Pinus taeda* L. *Genetics* **172**:1915-1926.
